# Reconfigurable Feedback Field-Effect Transistors with a Single Gate

**DOI:** 10.3390/nano13243133

**Published:** 2023-12-13

**Authors:** Yoocheon Lee, Doohyeok Lim

**Affiliations:** School of Electronic Engineering, Kyonggi University, Suwon 16227, Republic of Korea; leeyou0425@naver.com

**Keywords:** reconfigurable transistor, feedback field-effect transistor, positive feedback mechanism

## Abstract

In this study, we present a reconfigurable feedback field-effect transistor (FET) that can operate in both p- and n-type configurations using a feedback mechanism. In contrast to previously reported reconfigurable FETs, our device utilizes a single gate to trigger a feedback mechanism at the center, resulting in steep switching characteristics. The device exhibited high symmetry of transfer characteristics, an on/off current ratio of approximately 10^10^, extremely low subthreshold swings, and a high on-current of approximately 1.5 mA at low gate voltages in both configurations. In addition, because of their hysteresis and bistable characteristics, they can be applied to various electronic devices. These characteristics were analyzed using a commercial device simulator.

## 1. Introduction

For decades, the continuous scaling down of the complementary metal–oxide–semiconductor (CMOS) technology has led to revolutionary developments in information technology because of Moore’s Law [1], which states that the density of microchips doubles every 24 months. However, MOS field-effect transistors (MOSFET) encounter limitations in scaling down because of leakage current caused by phenomena such as the short-channel effect [2]. In particular, due to the thermal limit of carriers, the subthreshold swing has a limit of 60 mV/dec at room temperature [3]. Various devices such as tunneling FET (TFET) [4,5,6,7] that use tunneling effect, impact ionization MOS (I-MOS) [8,9,10,11] that uses impact ionization, and feedback FET (FBFET) [12,13,14,15,16,17,18,19,20,21,22,23,24] that uses feedback phenomena have been studied to overcome these limitations.

FBFET exhibits steep switching characteristics using a positive feedback mechanism by modulating the potential barrier in a structure such as p-n-p-n. The FBFET that was first proposed [12] modulated the potential barrier by trapping charges in gate-sidewall spacers. However, due to the spacer region’s additional process and instability, structures have been proposed to dope the existing spacer region with heavy doping or replace it with an additional gate electrode [14,15]. These structures are relatively stable and can be p- and n-type reconfigured in a single device with an additional gate electrode [13]. However, additional gate voltage modulation of a four-terminal device structure is necessary for reconfiguration in the p- and n-type operation modes.

In this study, we proposed a reconfigurable FBFET that can be operated in p- and n-type modes by controlling the single-gate voltage modulation. Single-gate voltages allow the injection of holes (p-type) or electrons (n-type) for a positive feedback loop. Contrary to other reconfigurable FETs (RFETs) [25,26,27,28,29], which exhibit unipolar conduction for electrons and holes by hindering the injection of undesired charge carriers, reconfigurable FBFETs use electrons and holes to conduct current. Therefore, our device exhibited symmetrical characteristics for p- and n-type configurations.

## 2. Device Structure and Simulation

**Figure 1:**
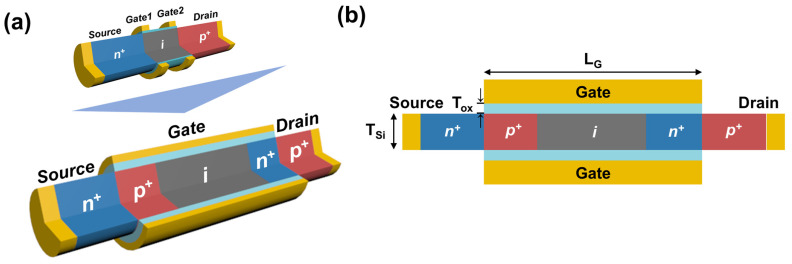
(**a**) Schematic illustration of the reconfigurable FBFET structure. (**b**) Cross-sectional view of the reconfigurable FBFET proposed in this study. A two-dimensional structure is used for simulation purposes.

Figure 1 shows the schematic of the reconfigurable FBFET structure. Unlike the previously reported reconfigurable FBFET with two separate gates [14], our reconfigurable FBFET utilizes a single gate to control hole and electron injections. We used Al_2_O_3_ with a dielectric constant of 9.3 [30] and thickness (T_ox_) of 3 nm for the gate oxide. The gate material used was platinum (Pt) with a work function of 5.65 eV, and a gate length (L_g_) of 60 nm was used for surrounding the channel; the length and thickness of the channel were 60 and 10 nm, respectively. We employed silicon, commonly utilized in CMOS processes, as the channel material. The gated channel region consisted of p-type, intrinsic, and n-type regions. We heavily doped the p- and n-types at 1 × 10^20^ cm^−3^ to replace the charge spacer [12,22,23]. The doping concentration of the lightly p-doped intrinsic region was 1 × 10^16^ cm^−3^ and of the n^+^ source and p^+^ drain region was 1 × 10^20^ cm^−3^, respectively.

We conducted our study with a two-dimensional structure for simulation purposes using a device simulator (Silvaco Atlas, version 5.2.17 R). The operation of our device is similar to that of a bipolar junction transistor (BJT) and includes a metal–oxide–semiconductor structure. Therefore, we used physical models, including concentration-dependent lifetime, Auger recombination, bandgap narrowing, field-dependent mobility, concentration-dependent mobility for BJT, and Lombardi Mobility models, for MOSFET [30]. In the simulation, the default parameters for these models were used. We analyzed the channel surface to obtain the energy band and simulated it using the transient ramp method.

## 3. Results and Discussion

**Figure 2:**
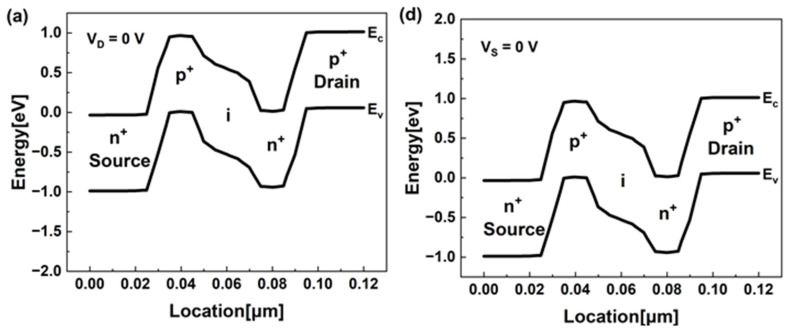

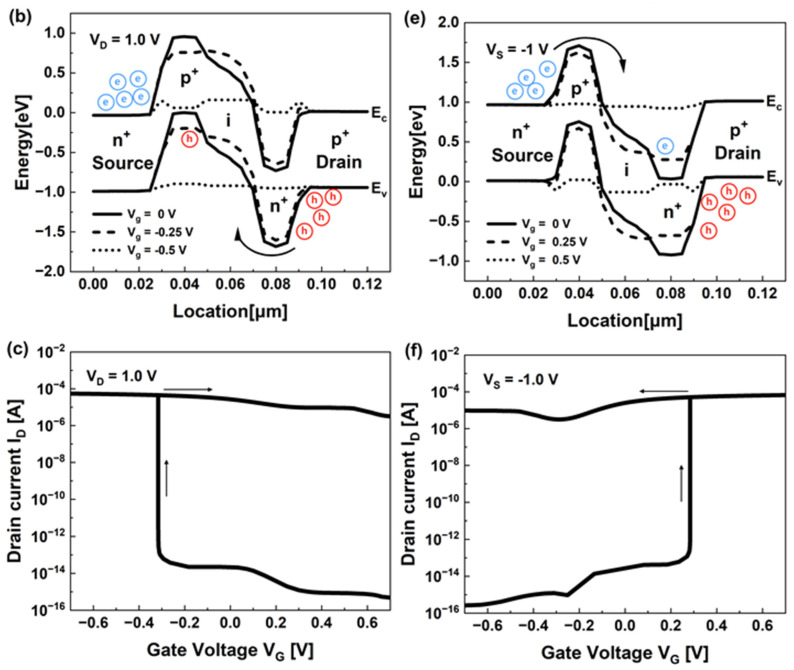
(**a**) Energy band diagrams along the semiconductor surface with no bias of p-configuration (V_D_ = 0 V, V_G_ = 0 V) and (**b**) different gate voltages of p-configuration causing positive feedback process (V_D_ = 1 V, V_G_ = 0, −0.25, −0.5 V). The symbols e/h in circle indicate electrons and holes, respectively. (**c**) Transfer characteristics caused by positive feedback (V_D_ = 1 V). The arrows indicate the sweeping directions. (**d**) Energy band diagrams along the semiconductor surface with no bias of n-configuration (V_S_ = 0 V, V_G_ = 0 V) and (**e**) different gate voltages of n-configuration causing positive feedback process (V_S_ = −1 V, V_G_ = 0, 0.25, 0.5 V). (**f**) Transfer characteristics caused by positive feedback process (V_S_ = −1 V)). The sweeping directions are indicated by the arrows.

Using a positive feedback mechanism, our reconfigurable FBFET can operate in both p- and n-type configurations in a single device. Figure 2a,d show an energy band diagram with no bias in off-state. We heavily doped both ends of the channel region adjacent to the p+ drain and n+ source regions to generate sufficient potential barriers. These potential barriers blocked the injection of charge carriers such as holes and electrons. Figure 2b shows the energy band diagrams of a process in which a positive feedback phenomenon occurs at a drain voltage of 1 V and the device turns on. If no voltage is applied to the gate, the potential barrier blocks the injection of charge carriers and is maintained in the off-state. When we applied a negative voltage to the gate, the energy levels of the intrinsic region and the valence band of the n^+^ region adjacent to the drain increased, and the potential barrier of the n^+^ region decreased. We injected holes in the drain region into the potential well of the p^+^ region adjacent to the source region, reducing the height of the potential barrier. Due to the reduced potential barrier, we injected electrons in the source region into the potential well near the drain region, further lowering the potential barrier. Because of the lower potential well, the injected holes accumulated more in the potential well and flowed into the source region as majority carrier currents. These phenomena occurred repeatedly, resulting in a positive feedback process; the potential barrier on both sides inside the channel region disappeared, and the device turned into an on-state abruptly. The phenomenon where a device suddenly turns on and significantly increases current is called the latch-up phenomenon.

Figure 2c shows the transfer characteristics depicted by the latch-up phenomenon. During the reverse sweep, the current increases rapidly with an extremely low subthreshold swing (SS), I_on_/I_off_ ratio of ~10^8^ near V_G_ = −0.31 V. When the gate voltage forward sweeps to 0.7 V, the device remains in an on-state. Similar to Figure 2b for the p-configuration, Figure 2e shows the energy band diagram of the n-configuration. When the source voltage is −1 V, the gate voltage increases, indicating the positive feedback phenomenon. As the gate voltage increased, the potential barrier of the p^+^ region adjacent to the source decreased, and the energy level of the intrinsic region decreased. Through the lowered potential barrier, we injected electrons into the potential well near the drain region, lowering the barrier height and injecting holes into the p^+^ region near the source. Thus, we lowered the potential barrier adjacent to the source, and this repeated positive feedback phenomenon caused a steep switching, as Figure 2f shows. In the case of the n configuration, latch-up occurred at V_G_ = 0.27 V and had a symmetrical transfer characteristic with p-configuration. When comparing the two cases, for the p-configuration, holes in the drain region are initially injected into the channel region as the gate voltage decreases, inducing the positive feedback phenomenon. Conversely, for the n-configuration, electrons in the source region are initially injected into the channel region as the gate voltage increases, resulting in the positive feedback phenomenon. Consequently, the device can be reconfigured between p- and n-configurations by adjusting the gate voltage based on the applied drain/source voltages.

**Figure 3:**
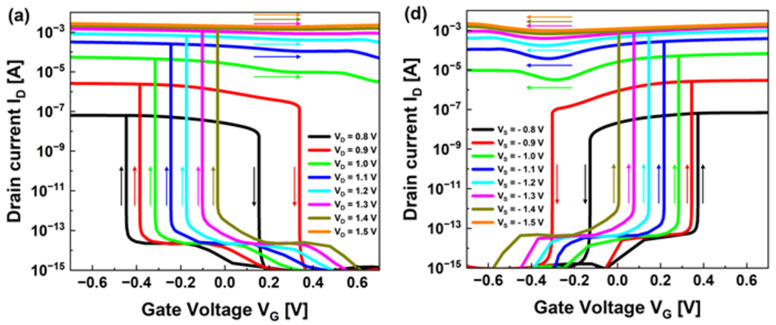

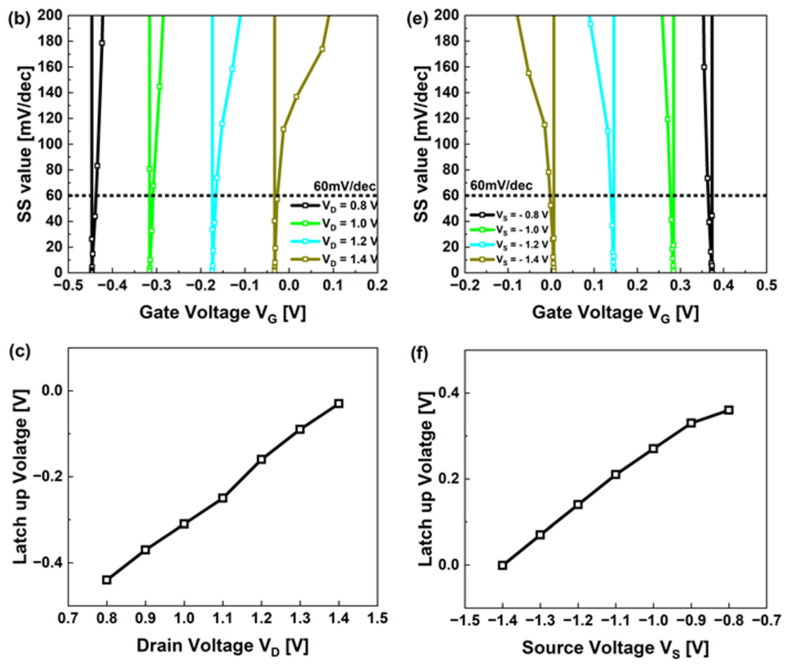
Electrical characteristics of the reconfigurable FBFET. (**a**) Transfer characteristics for the p-configuration with various drain voltages. The arrows indicate the sweeping directions. (**b**) SS values as a function of gate voltage with different drain voltages. (**c**) Latch-up voltage values determined by the transfer curve for the p-configuration. (**d**) Transfer characteristics for the n-configuration with various source voltages. The sweeping directions are indicated by the arrows. (**e**) SS values as a function of gate voltage with different source voltages. (**f**) Latch-up voltage values determined by the transfer curve for the n-configuration.

To investigate the various properties of our device, Figure 3a shows the transfer characteristics of the p-configuration at various drain voltages. When V_D_ is 0.8–0.9 V and if V_G_ is swept from 0.7 V to −0.7 V, a latch-up occurs at V_G_ = −0.44 V and −0.37 V. When V_G_ is forward swept to 0.7 V, a latch-down occurs at V_G_ = 0.15 V and 0.33 V, generating a clockwise hysteresis loop because of the excessive charge carriers in the channel region after the positive feedback process. Excessive charge carriers regenerate the potential barrier, and the current rapidly decreases at a latch-down voltage that is different from the latch-up voltage. When V_D_ is between 1 V and 1.4 V, a latch-up occurs between approximately −0.31 V and 0 V if the gate voltage is reverse swept. However, during a forward voltage sweep in that state, it does not latch down and remains in the on-state, exhibiting bistable characteristics, meaning that increasing the gate voltage cannot generate a sufficient barrier height; thus, the positive feedback phenomenon continues to occur. When V_D_ = 1.4 V, the on/off current ratio is approximately 10^10^ and has a high on current (~1.5 mA). If V_D_ exceeds 1.5 V, the device does not exhibit switching characteristics and remains in the state because, due to the excessive voltage from the drain, the potential barrier of the drain region is not sufficiently generated to block the injection of the hole. The transfer curve’s Subthreshold Swing (SS) at various drain voltages is plotted in Figure 3b. SS extracts the vicinity of the latch-up section in Figure 3a and defines it as [d(log I_D_)/dV_G_]^−1^. Our device exhibits a steep switching characteristic close to 0 mV/dec by solving the limit of 60 mV/dec of MOSFET at room temperature. For V_D_ of 0.8, 1.0, 1.2, and 1.4 V, the minimum SS values are 3.1 × 10^−3^, 4.13 × 10^−7^, 6.24 × 10^−7^, and 8.18 × 10^−8^ mV/dec, showing better-switching characteristics as the drain voltage increases due to the reduced potential barrier in the drain region. Figure 3c shows the latch-up voltage of the transfer curve for various drain voltages. The latch-up voltage is defined based on when the SS value is less than 60 mV/dec. As the voltage applied to the drain increases by 0.1 V, the potential barrier in the drain region constantly decreases. The gate voltage required for constant latch-up to occur changes (−0.44 → −0.37 → −0.31 → −0.25 → −0.16 → −0.09 → −0.03 V), and Figure 3c shows a linear characteristic.

Figure 3d shows the transfer characteristics of the n-configuration under various source voltages symmetric to the p-configuration. When V_S_ is −0.8 V to −0.9 V, counterclockwise hysteresis characteristics appear if V_G_ forward sweeps to 0.7 V and reverse sweeps to −0.7 V. In this section, as V_S_ increases (−0.8 V → −0.9 V), the hysteresis window’s value also increases (0.5 V → 0.64 V). Similarly, when V_S_ is between −1 V and −1.4 V, the device does not turn off after a rapid increase in current. As the drain bias increases, the on-current also increases because as the drain bias increases, the electric field in the channel region increases, accelerating the carriers and speeding them up. When V_S_ is −1.4 V, it exhibits good switching characteristics (on/off current ratio of ~10^10^, on current of ~1.5 mA). When V_S_ is −1.5 V, the device remains in the on-state with no switching characteristics. The SS and latch-up voltages according to the various source voltages in Figure 3d are shown in Figure 3e and Figure 3f, respectively. Both values are defined similarly as in the p-configuration and exhibit symmetric characteristics with the p-configuration. As the fixed source voltage changes by −0.2 V from −0.8 V to −1.4 V, the minimum SS values are 8.84 × 10^−5^, 2.98 × 10^−7^, 6.82 × 10^−7^, and 5.9 × 10^−8^ mV/dec, gradually approaching 0 mV/dec. In the case of the latch-up voltage, it increases relatively constantly as it changes by −0.1 V from −0.8 V to −1.4 V (0.36 → 0.33 → 0.27 → 0.21 → 0.14 → 0.07 → −0.001 V). There is a slight difference in the symmetry of the latch-up voltage between the p- and n-configurations due to the difference in the electron and hole mobilities. Despite these differences, our device provides numerous benefits when applied to logic devices because of its nearly symmetrical reconfigurable characteristics.

**Figure 4:**
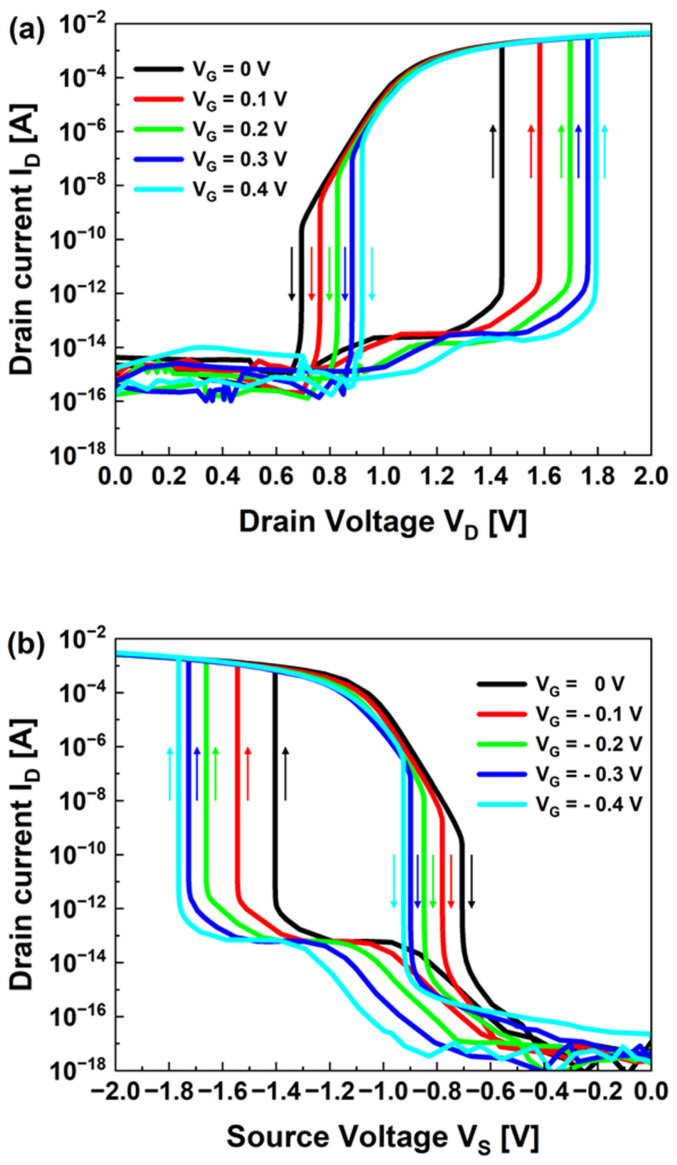
Hysteresis characteristics in the output curves with various gate voltages for the (**a**) p-configuration and (**b**) n-configuration. The arrows indicate the sweeping directions.

Next, we analyzed the output curves for both configurations. In the p-configuration (Figure 4a), the potential barrier height near the drain region increased as the gate voltage increased. Because of the increased barrier height, we required a higher drain voltage to inject holes into the potential well near the source region. When the fixed gate voltage increased from 0–0.4 V when sweeping the drain forward, the latch-up voltage increased. When reverse sweeping after the latch-up phenomenon, the latch-up and latch-down voltages differed, forming a counterclockwise hysteresis loop.

Similarly, the output curve of the n-configuration in Figure 4b, which causes a positive feedback process due to electron injection, shows clockwise hysteresis. The height of the potential barrier in the source region varied according to the fixed gate voltage. The source voltage required for electron injection and the latch-up voltage changed. Each hysteresis loop had a constant width as the gate voltage changed. The width of the hysteresis loop varied with the degree and type of charge carrier injection. When comparing the output characteristics of the p- and n-configurations, they exhibited mostly symmetrical characteristics except for the off-current. The higher off-current in the p-configuration compared to the n-configuration is the lower mobility of holes, which makes them more likely to become trapped in the gate oxide layer or channel region, resulting in an increased leakage current.

Table 1 compares the electrical characteristics of the devices available in one device, both p- and n-types, using the feedback phenomenon from one device. Most RFET [25,28,29] using Schottky barrier tunneling (SBT) have asymmetric characteristics due to the tunneling probability and mobility of electrons and holes. In contrast, devices using a positive feedback mechanism have symmetrical characteristics because electrons and holes conduct current [14]. Unlike the devices in Table 1, our device has a single gate functioning as the program and control gates. This single-gate FBFET does not require the precise alignment and patterning of the two separate gates. We reduced the number of process steps, reducing costs and improving productivity. As the potential barriers play a critical role in the positive feedback process, channel length affects the gain of the positive feedback loop. As the channel length becomes shorter, less charge is required in the potential well in order to lower the height of the potential barrier. Since the increased gain of the positive feedback loop leads to an increase in electrical performance, our device exhibits excellent switching characteristics due to its short channel length. Therefore, the simplicity and enhanced switching performance of the proposed single-gate reconfigurable FBFET makes it a promising candidate for various applications, including low-power electronics, memory devices, and logic devices.

## 4. Conclusions

In this study, we proposed a reconfigurable FBFET using a single gate. We applied the p- and n-type configurations to a single device by modulating the height of the barrier using a single gate. Unlike the previously reported FBFET and tunneling FETs with two separate gates [14,31,32,33,34], the single gate of our reconfigurable FBFET can control hole and electron injections for p- and n-configurations, respectively. Based on the positive feedback, it had excellent switching characteristics, including an on/off current ratio of ~10^10^ and an SS close to 0 mV/dec. In addition to symmetric characteristics in the p- and n-type configurations through a symmetrical structure, our device can be applied to various fields such as reconfigurable logic-in-memory.

## Figures and Tables

**Table 1:** Comparison of the electrical characteristics of different reconfigurable devices using positive-feedback mechanism.

Ref.	Features	Channel Size	SS min (mV/dec)		Ion		Ion/Ioff		Year
			p	n	p	n	p	n	
[13]	Si NW, plastic substrate, dual top gate, bending compatible	L = 5 μm H = 150 nm	10	18	~10^−5^		~10^6^		2015
[14]	p^+^-i-n^+^ with two separate gates, SiNW FET	L = 4 μm W = 30 nm	1.78	1.36	~10^−3^		~10^5^		2019
[24]	p^+^-i-n^+^ with two separate gates (program gate, control gate), SINW FET	L = 200 nm W(T_si_) = 12 nm	0.4		~10^−6^		~10^12^		2022
this work	n^+^-p^+^-i-n^+^-p^+^ with single gate, SINW FET	L = 60 nm W(T_si_) = 10 nm	8.2 × 10^−8^	5.9 × 10^−8^	~1.5 × 10^−3^		~10^10^		2023

## Data Availability

All data generated or analyzed in this study are included in the published article. The datasets used and/or analyzed in the current study are available from the corresponding author upon reasonable request.

## References

[1] Moore G.E. (2006). Cramming More Components onto Integrated Circuits, Reprinted from Electronics, Volume 38, Number 8, April 19, 1965, Pp.114 Ff. IEEE Solid-State Circuits Soc. Newsl..

[2] Khanna V.K. (2016). Short-Channel Effects in MOSFETs BT—Integrated Nanoelectronics: Nanoscale CMOS, Post-CMOS and Allied Nanotechnologies.

[3] Lim D., Kim M., Kim Y., Kim S. (2017). Memory Characteristics of Silicon Nanowire Transistors Generated by Weak Impact Ionization. Sci. Rep..

[4] Knoch J., Mantl S., Appenzeller J. (2007). Impact of the Dimensionality on the Performance of Tunneling FETs: Bulk versus One-Dimensional Devices. Solid State Electron..

[5] Datta S., Liu H., Narayanan V. (2014). Tunnel FET Technology: A Reliability Perspective. Microelectron. Reliab..

[6] Boucart K., Ionescu A.M. (2007). Double-Gate Tunnel FET with High-k Gate Dielectric. IEEE Trans. Electron Devices.

[7] Kim J.H., Kim S., Park B.-G. (2019). Double-Gate TFET with Vertical Channel Sandwiched by Lightly Doped Si. IEEE Trans. Electron Devices.

[8] Choi W.Y., Song J.Y., Lee J.D., Park Y.J., Park B.-G. (2005). A Novel Biasing Scheme for I-MOS (Impact-Ionization MOS) Devices. IEEE Trans. Nanotechnol..

[9] Gopalakrishnan K., Griffin P.B., Plummer J.D. I-MOS: A Novel Semiconductor Device with a Subthreshold Slope Lower than KT/Q. Proceedings of the Digest, International Electron Devices Meeting.

[10] Hoeneisen B., Mead C.A. (1972). Fundamental Limitations in Microelectronics—I. MOS Technology. Solid State Electron..

[11] Choi W.Y., Song J.Y., Lee J.D., Park Y.J., Park B.-G. (2005). 100-Nm n-/p-Channel I-MOS Using a Novel Self-Aligned Structure. IEEE Electron Device Lett..

[12] Padilla A., Yeung C.W., Shin C., Hu C., Liu T.-J.K. Feedback FET: A Novel Transistor Exhibiting Steep Switching Behavior at Low Bias Voltages. Proceedings of the Electron Devices Meeting.

[13] Jeon Y., Kim M., Lim D., Kim S. (2015). Steep Subthreshold Swing N- and p-Channel Operation of Bendable Feedback Field-Effect Transistors with P+–i–N+ Nanowires by Dual-Top-Gate Voltage Modulation. Nano Lett..

[14] Lim D., Kim S. (2019). Polarity Control of Carrier Injection for Nanowire Feedback Field-Effect Transistors. Nano Res..

[15] Choi S., Son J., Cho K., Kim S. (2021). One-Transistor Static Random-Access Memory Cell Array Comprising Single-Gated Feedback Field-Effect Transistors. Sci. Rep..

[16] Wan J., Royer C.L., Zaslavsky A., Cristoloveanu S. (2012). A Compact Capacitor-Less High-Speed DRAM Using Field Effect-Controlled Charge Regeneration. IEEE Electron Device Lett..

[17] Yang Y., Park Y.-S., Son J., Cho K., Kim S. (2021). Simulation Studies on Electrical Characteristics of Silicon Nanowire Feedback Field-Effect Transistors with Interface Trap Charges. Sci. Rep..

[18] Lim D., Son J., Cho K., Kim S. (2020). Quasi-Nonvolatile Silicon Memory Device. Adv. Mater. Technol..

[19] Lim D., Cho K., Kim S. (2022). Reconfigurable Logic-in-Memory Using Silicon Transistors. Adv. Mater. Technol..

[20] Wan J., Royer C.L., Zaslavsky A., Cristoloveanu S. (2013). A Systematic Study of the Sharp-Switching Z2-FET Device: From Mechanism to Modeling and Compact Memory Applications. Solid State Electron..

[21] Solaro Y., Wan J., Fonteneau P., Fenouillet-Beranger C., Le Royer C., Zaslavsky A., Ferrari P., Cristoloveanu S. (2014). Z2-FET: A Promising FDSOI Device for ESD Protection. Solid State Electron..

[22] Yeung C.W., Padilla A., Liu T.-J.K., Hu C. Programming Characteristics of the Steep Turn-on/off Feedback FET (FBFET). Proceedings of the 2009 Symposium on VLSI Technology.

[23] Jeon Y., Kim M., Kim Y., Kim S. (2014). Switching Characteristics of Nanowire Feedback Field-Effect Transistors with Nanocrystal Charge Spacers on Plastic Substrates. ACS Nano.

[24] Jeon J., Woo S., Cho K., Kim S. (2022). Logic and Memory Functions of an Inverter Comprising Reconfigurable Double Gated Feedback Field Effect Transistors. Sci. Rep..

[25] Heinzig A., Slesazeck S., Kreupl F., Mikolajick T., Weber W.M. (2012). Reconfigurable Silicon Nanowire Transistors. Nano Lett..

[26] Simon M., Liang B., Fischer D., Knaut M., Tahn A., Mikolajick T., Weber W.M. (2020). Top-Down Fabricated Reconfigurable FET with Two Symmetric and High-Current On-States. IEEE Electron Device Lett..

[27] Weber W.M., Heinzig A., Trommer J., Martin D., Grube M., Mikolajick T. (2014). Reconfigurable Nanowire Electronics—A Review. Solid State Electron..

[28] Trommer J., Heinzig A., Mühle U., Löffler M., Winzer A., Jordan P.M., Beister J., Baldauf T., Geidel M., Adolphi B. (2017). Enabling Energy Efficiency and Polarity Control in Germanium Nanowire Transistors by Individually Gated Nanojunctions. ACS Nano.

[29] Glassner S., Zeiner C., Periwal P., Baron T., Bertagnolli E., Lugstein A. (2014). Multimode Silicon Nanowire Transistors. Nano Lett..

[30] Silvaco Inc (2016). Atlas User’s Manual.

[31] Singh G., Amin S.I., Anand S., Sarin R.K. (2016). Design of Si0.5Ge0.5 Based Tunnel Field Effect Transistor and Its Performance Evaluation. Superlattices Microstruct..

[32] Anam A., Amin S.I., Prasad D., Kumar N., Anand S. (2023). Charge-Plasma-Based Inverted T-Shaped Source-Metal Dual-Line Tunneling FET with Improved Performance at 0.5 V Operation. Phys. Scr..

[33] Singh S., Raman A. (2018). Gate-All-Around Charge Plasma-Based Dual Material Gate-Stack Nanowire FET for Enhanced Analog Performance. IEEE Trans. Electron Devices.

[34] Anand S., Amin S.I., Sarin R.K. (2016). Analog Performance Investigation of Dual Electrode Based Doping-Less Tunnel FET. J. Comput. Electron..

